# The association between malnutrition and the incidence of malaria among young HIV-infected and -uninfected Ugandan children: a prospective study

**DOI:** 10.1186/1475-2875-11-90

**Published:** 2012-03-27

**Authors:** Emmanuel Arinaitwe, Anne Gasasira, Wendy Verret, Jaco Homsy, Humphrey Wanzira, Abel Kakuru, Taylor G Sandison, Sera Young, Jordan W Tappero, Moses R Kamya, Grant Dorsey

**Affiliations:** 1 Makerere University-University of California, San Francisco Research Collaboration, Mulago Hospital Complex, PO Box 7475, Kampala, Uganda; 2 Department of Epidemiology, University of California, Berkeley, CA, USA; 3 Global Health Sciences, University of California, San Francisco, CA, USA; 4 Department of Medicine, University of Washington, Seattle, WA, USA; 5 Division of Nutritional Sciences, Cornell University, Ithaca, NY, USA; 6 Center for Global Health, Centers for Disease Control and Prevention, Atlanta, GA, USA; 7 Department of Medicine, Makerere University College of Health Sciences, Kampala, Uganda; 8 Department of Medicine, University of California, San Francisco, CA, USA

## Abstract

**Background:**

In sub-Saharan Africa, malnutrition and malaria remain major causes of morbidity and mortality in young children. There are conflicting data as to whether malnutrition is associated with an increased or decreased risk of malaria. In addition, data are limited on the potential interaction between HIV infection and the association between malnutrition and the risk of malaria.

**Methods:**

A cohort of 100 HIV-unexposed, 203 HIV-exposed (HIV negative children born to HIV-infected mothers) and 48 HIV-infected children aged 6 weeks to 1 year were recruited from an area of high malaria transmission intensity in rural Uganda and followed until the age of 2.5 years. All children were provided with insecticide-treated bed nets at enrolment and daily trimethoprim-sulphamethoxazole prophylaxis (TS) was prescribed for HIV-exposed breastfeeding and HIV-infected children. Monthly routine assessments, including measurement of height and weight, were conducted at the study clinic. Nutritional outcomes including stunting (low height-for-age) and underweight (low weight-for-age), classified as mild (mean z-scores between -1 and -2 during follow-up) and moderate-severe (mean z-scores < -2 during follow-up) were considered. Malaria was diagnosed when a child presented with fever and a positive blood smear. The incidence of malaria was compared using negative binomial regression controlling for potential confounders with measures of association expressed as an incidence rate ratio (IRR).

**Results:**

The overall incidence of malaria was 3.64 cases per person year. Mild stunting (IRR = 1.24, 95% CI 1.06-1.46, p = 0.008) and moderate-severe stunting (IRR = 1.24, 95% CI 1.03-1.48, p = 0.02) were associated with a similarly increased incidence of malaria compared to non-stunted children. Being mildly underweight (IRR = 1.09, 95% CI 0.95-1.25, p = 0.24) and moderate-severe underweight (IRR = 1.12, 95% CI 0.86-1.46, p = 0.39) were not associated with a significant difference in the incidence of malaria compared to children who were not underweight. There were no significant interactions between HIV-infected, HIV-exposed children taking TS and the associations between malnutrition and the incidence of malaria.

**Conclusions:**

Stunting, indicative of chronic malnutrition, was associated with an increased incidence of malaria among a cohort of HIV-infected and -uninfected young children living in an area of high malaria transmission intensity. However, caution should be made when making causal inferences given the observational study design and inability to disentangle the temporal relationship between malnutrition and the incidence of malaria.

**Trial Registration:**

ClinicalTrials.gov: NCT00527800.

## Background

Malaria is a leading cause of childhood morbidity and mortality in sub-Saharan Africa [1]. According to the World Health Organization, in 2008 there were an estimated 243 million cases of malaria and 863,000 deaths of which 89% occurred in Africa and 88% of African deaths occurred in children under five years of age [2]. Malnutrition is also a major public health problem in African children, with estimated 38% stunted, 28% underweight, and 9% wasted [3]. Malnutrition is responsible for an increased risk of a number of common childhood infectious diseases including diarrhoea and upper respiratory tract infections [4,5], as well as increased case fatality [4,6].

Findings from studies evaluating associations between various measures of malnutrition and malaria have been contradictory. Some studies suggest a protective effect against malaria for wasted children [7] and children with stunting [8,9]. In contrast, other studies have found either no association between various definitions of malnutrition and the risk of malaria [10-12], or an increased risk of malaria among stunted [13] and underweight children [14]. Comparisons across these studies are complicated by differences in study design, definitions of malnutrition and malaria morbidity, patient populations, and local malaria epidemiology.

The role that HIV exposure and infection may have on potential associations between malnutrition and malaria in children has not yet been reported. This is a critical gap because HIV infection has been associated with an increased risk of both malnutrition [15,16] and malaria [17-20]. Further, because chemoprophylaxis with trimethoprim-sulphamethoxazole (TS) has become the standard of care for breastfeeding HIV-exposed infants (HIV-negative children born to HIV-infected mothers) and all HIV-infected children, and chemoprophylaxis with TS is protective against malaria [21,22]. Such an intervention may further modulate any potential interaction between HIV and associations between malnutrition and malaria.

To assess associations between paediatric malnutrition and the incidence of malaria by HIV status, prospective data were collected from a cohort of HIV-unexposed, HIV-exposed and HIV-infected children living in Tororo, a rural area in Uganda with high malaria transmission intensity [23].

## Methods

### Study site and participants

This study was conducted in the district of Tororo, a rural area in south-eastern Uganda. Malaria transmission in Tororo is holo-endemic, occurring perennially with an entomologic inoculation rate estimated to be 562 infective bites per person-year [23]. Data were collected from a cohort of HIV-unexposed, HIV-exposed, and HIV-infected children as has been described elsewhere [24]. Briefly, convenience sampling was used to enrol children referred to a dedicated study clinic from an adjacent post-natal clinic at Tororo District Hospital. Eligibility criteria included: 1) age six weeks to 12 months; 2) documented HIV status of mother and child by DNA polymerase chain reaction (PCR); 3) agreement to come to the study clinic for any febrile episode or other illness; 4) residence within a 30 km radius of the study clinic; 5) absence of active medical problem requiring in-patient evaluation at the time of screening; 6) provision of informed consent; and 7) currently breastfeeding if HIV-exposed. At enrolment, all study participants received long-lasting insecticide-treated bed nets (ITN) and all HIV-exposed and HIV-infected children were given daily TS prophylaxis as per Uganda's Ministry of Health (MOH) guidelines. HIV-unexposed children were not prescribed TS prophylaxis. The study protocol was approved by the Uganda National Council of Science and Technology and the institutional review boards of the University of California, San Francisco, Makerere University, the University of Washington, and the US Centers for Disease Control and Prevention.

### Follow-up of study participants

Subjects were followed for all medical problems at a dedicated study clinic open seven days a week from 8 am to 5 pm. Parents (or guardians) were encouraged to bring their children to the study clinic whenever they were ill. After-hours care was available through the Tororo District Hospital. Children who presented with new medical problems underwent a standardized medical evaluation using algorithms to guide therapy for common illnesses that were developed locally by the study investigators. Medications with anti-malarial activity were avoided for the treatment of non-malarial illnesses. Monthly routine assessments were performed in the study clinic to ensure adherence with the study protocol. HIV-exposed children were re-tested for HIV by DNA PCR six to eight weeks after breastfeeding cessation. Those children who remained HIV-uninfected after breastfeeding cessation were then randomized to continue or discontinue TS prophylaxis as has been described elsewhere [25]. HIV-infected children, including those who seroconverted during breastfeeding, were continued on TS prophylaxis for the duration of the study. All HIV-infected children were anti-retroviral therapy (ART) naïve at enrolment and ART (nevirapine + lamivudine + zidovudine or stavudine) was initiated for those participants who met standardized WHO criteria. Study participants' compliance to TS, ART and ITN was assessed through questionnaires administered to their mothers at monthly visits. Study participants were not provided supplemental feeding. Study participants were withdrawn from the study for: 1) movement out of the study area; 2) inability to be located for > 60 consecutive days; 3) withdrawal of informed consent; 4) inability to adhere to the study schedule and procedures; or 5) inability to tolerate the drugs used for malaria treatment.

### Anthropometric measurements

Weight and length/height measurements were taken at every visit to the study clinic at least once a month by trained study nurses in accordance with internationally recommended procedures [26]. Participants were weighed using a designated baby scale (Seca- 310, Hamburg, Germany) precise to the nearest 0.1 kg. Recumbent length measurements precise to the nearest centimetre were taken for children < 1 year of age using a stadiometer board and standing height was measured for older children precise to the nearest centimetre using TALC height measuring chart (Teaching Aids at Low Cost, St Albans, UK).

### Malaria diagnosis and management

Subjects who presented to the study clinic with a documented fever (tympanic temperature ≥ 38.0°C) or history of fever in the previous 24 hours, had blood obtained by finger prick for a thick smear. If the thick smear was positive, the patient was diagnosed with malaria regardless of parasite density. Study participants ≥ 4 months and ≥ 5 kg were randomly assigned to receive either open label artemether-lumefantrine (AL) or dihydroartemisinin-piperaquine (DP) at the time their first episode of uncomplicated malaria was diagnosed. Study participants received the same treatment regimen for all subsequent episodes of uncomplicated malaria. Episodes of uncomplicated malaria in children < 4 months of age or weighing < 5 kg, as well as episodes of complicated malaria and treatment failures occurring within 14 days of initiating treatment were treated with quinine.

### Laboratory methods

Thick and thin blood smears were stained with 2% Giemsa for 30 minutes. A thick smear was considered negative if no parasites were seen after review of 100 high-powered fields. Speciation of parasites was based on readings of thin smears by a trained microscopist. A final diagnosis of malaria used in the analysis was based on a rigorous quality-control system that included re-reading of all blood smears by a second microscopist and resolution of any discrepancies between the first and second readings by a third microscopist within 24 hours of the initial smear reading.

Assessment of infants HIV status prior to enrolment and at six to eight weeks after cessation of breastfeeding involved collecting a heel, toe or finger-prick blood sample on filter paper which was sent to a reference laboratory for HIV DNA PCR using Roche Amplicor HIV-1 DNA PCR test v1.5 kits (Roche Diagnostic Systems, Inc., Branchburg, New Jersey, USA).

### Statistical analysis

Data were double entered in Access (Microsoft Corporation, Redmond, Washington, USA), and statistical analysis was performed using STATA, version 10 (Stata Corporation, College Station, Texas, USA). The observation period for each study participant began the day after enrolment and ended when a study participant was either prematurely withdrawn from the study or reached 2.5 years of age.

Nutritional status was assessed using the following standardized anthropometric z-scores as per WHO guidelines [27,28]: height-for-age z- score (HAZ) for stunting and weight-for-age z-score (WAZ) for underweight. Measures of malnutrition were categorized as mild (z-score between -1 and -2) and moderate-severe (z-score < -2). Wasting (based on weight-for-height z-scores) was not included as a measure of nutritional status because values < -1 were relatively uncommon and therefore lacked statistical power for analyses. Repeated cross-sectional associations between variables of interest (breastfeeding, HIV-status, use of TS chemoprophylaxis and location of residence) and measures of malnutrition (dichotomized using z-scores < -1 as a cut-off and expressed) were estimated as a relative risk using generalized estimating equations with exchangeable correlation and robust standard error, binomial distribution, and adjustment for repeated measures within individuals.

Malaria incidence was defined as the number of new episodes of malaria per time at risk. Episodes of malaria occurring within 14 days of a previous episode were not considered incident events. Longitudinal associations between variables of interest and malaria incidence were estimated as an incidence rate ratio using generalized estimating equations with exchangeable correlation, negative binomial distribution, and adjusted for repeated measures. For longitudinal associations, measures of malnutrition were categorized into none, mild, and moderate-severe based on the cut-offs above for mean z-scores during observation periods of interest. Interaction among potential confounders was also examined by including interaction terms into the multivariate models. A p-value < 0.05 was considered statistically significant for all analyses.

## Results

### Study profile and characteristics of study participants

**Figure 1:**
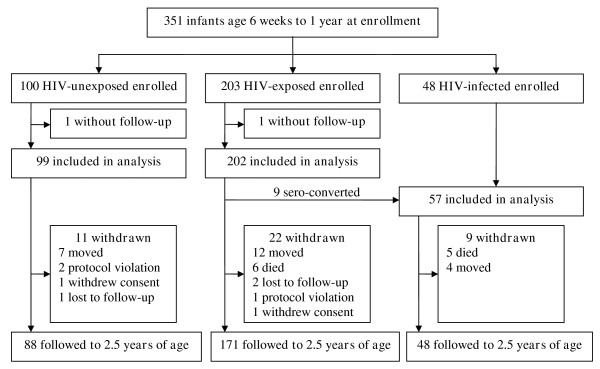
**Study profile**.

A total of 100 HIV-unexposed children, 203 HIV-exposed children, and 48 HIV-infected children were enrolled between August 2007 and April 2008 (Figure 1). Two participants with no follow-up information were not included in this analysis. During follow-up, nine HIV-exposed children seroconverted during breastfeeding resulting in a total of 57 HIV-infected children. Of the 349 study participants included in the study, a total of 307 (88%) reached 2.5 years of age with the remaining 42 prematurely withdrawn.

Study participants were followed for mean of 16 months, resulting in a total observation period of 662 person-years. HIV-exposed children were prescribed TS for a total of 73% of the observation period (Table 1). Mothers of the HIV-infected and HIV-exposed children prescribed TS reported that their children took > 99% of their doses. HIV-unexposed children breastfed until a median age of 20 months compared to 17 months among the HIV-infected children and seven months among the HIV-exposed children, whose mothers were counseled to rapidly wean after six months of age (per national recommendations at the time). During routine follow-up visits, mothers reported that over 98% of children slept under an ITN the previous night. All 57 HIV-infected children were ART naïve at enrolment. Fifty-two (91%) were initiated on ART, two (3.5%) were eligible but withdrew from the study before ART could be initiated, and three (5.2%) never met eligibility criteria for ART initiation.

**Table 1:** Characteristics of study participants

Characteristic	Risk group		
	
	HIV-unexposed (n = 99)	HIV-exposed (n = 202)	HIV-infected (n = 57)
Female gender, n (%)	40 (40%)	101 (50%)	28 (49%)

Residence in urban area, n (%)	23 (23%)	39 (19%)	18 (32%)

Median duration of follow-up, months (IQR)	15.2 (12.1-17.5)	16.7 (13.4-19.5)	14.3 (11.8-18.4)

Mean age during follow-up, months (SD)	14.3 (2.0)	13.2 (2.8)	14.3 (3.1)

Proportion of follow-up time prescribed TS	0	73%	100%

Proportion of follow-up time breastfeeding	81%	24%	64%

Mean HAZ scores			

Mild stunting, n (%)	38 (38%)	71 (35%)	17 (30%)

Moderate-severe stunting, n (%)	28 (28%)	69 (34%)	29 (51%)

Mean WAZ scores			

Mild underweight	23 (23%)	52 (26%)	13 (23%)

Moderate-severe underweight	7 (7.1%)	21 (10%)	17 (30%)

### Variables associated with measures of malnutrition

**Figure 2:**
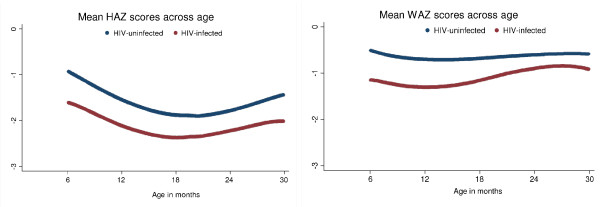
**Relationship between mean height-for-age Z scores (HAZ) and weight-for-age Z scores (WAZ) across age stratified by HIV status (using lowess smoothing)**.

Overall, 36% of study participants were mildly stunted and 34% were moderate-severely stunted. In contrast, 25% of study participants were mildly underweight and 13% were moderate-severely underweight. Mean HAZ scores decreased from six to 18 months of age (the age at which most children were stopping breastfeeding) and then rose slightly to 30 months of age (Figure 2). HAZ scores were consistently lower for HIV-infected children compared to HIV-uninfected children at all ages (Figure 2). Mean WAZ scores were fairly constant from six to 30 months of age and lower for HIV-infected children, particularly in younger children.

In multi-variate analysis, both breastfeeding (RR = 0.75, 95% CI 0.69-0.82, p < 0.001) and being prescribed TS chemoprophylaxis (RR = 0.82, 95% CI 0.74-0.90, p < 0.001) were associated with a lower risk of stunting of any severity (Table 2). HIV-infection was associated with a higher risk of stunting (RR = 1.38, 95% CI 1.18-1.62, p < 0.001). The only significant risk factor for being underweight of any severity was HIV-infection (RR = 1.41, 95% CI 1.02-1.96, p < 0.001).

**Table 2:** Variables associated with measures of malnutrition

Variable	Proportion with z-score < -1		Unadjusted RR* (95% CI)	p-value	Adjusted RR** (95% CI)	p-value
					
	Exposed group^†^	Unexposed group				
Stunting (HAZ-score)						

Breastfeeding	52%	73%	0.70 (0.64-0.76)	< 0.001	0.75 (0.69-0.82)	< 0.001

HIV-infected	79%	62%	1.34 (1.16-1.54)	< 0.001	1.38 (1.18-1.62)	< 0.001

TS chemoprophylaxis	59%	71%	0.78 (0.71-0.85)	< 0.001	0.82 (0.74-0.90)	< 0.001

Urban vs. rural residence	57%	65%	0.87 (0.73-1.04)	0.12	0.87 (0.74-1.03)	0.10

Being underweight (WAZ-score)						

Breastfeeding	34%	37%	0.89 (0.78-1.01)	0.08	0.89 (0.78-1.02)	0.09

HIV infected	49%	34%	1.48 (1.09-2.01)	0.01	1.41 (1.02-1.96)	0.04

TS chemoprophylaxis	37%	34%	1.06 (0.90-1.25)	0.49	1.06 (0.89-1.27)	0.52

Urban vs. rural residence	33%	36%	0.91 (0.66-1.26)	0.57	0.88 (0.65-1.21)	0.45

^† ^Exposed groups include breastfeeding, HIV infected participants, TS chemoprophylaxis, and urban residence

*RR relative risk using univariate analysis including only the variable of interest

**RR relative risk using multivariate analysis including all variables of interest listed in the table

### Variables associated with the incidence of malaria

By the end of the observation period, 288 (83%) of the study participants had experienced at least one episode of malaria. A total of 2,414 new episodes of malaria were diagnosed, resulting in an overall incidence of 3.64 episodes per person year. In multi-variate analysis, children with mild stunting (IRR = 1.24, 95% CI 1.06-1.46, p = 0.008) and moderate-severe stunting (IRR = 1.24, 95% CI 1.03-1.48, p = 0.02) had a similarly higher incidence of malaria compared to children without stunting (Table 3). The association between stunting and an increased incidence of malaria was similar for HIV-infected and uninfected children (p = 0.52 for interaction term in multi-variate model). As expected, being prescribed TS prophylaxis and living in an urban residence were associated with a significantly lower incidence of malaria. Notably, breastfeeding was associated with a lower incidence of malaria in the uni-variate analysis, but not in the multi-variate analysis. Increasing age was associated with a significantly higher incidence of malaria. On the other hand, HIV infection was not associated with the incidence of malaria in the multi-variate analysis (Table 3). In contrast, being mildly underweight (IRR = 1.09, 95% CI 0.95-1.25, p = 0.24) and moderate-severe underweight (IRR = 1.12, 95% CI 0.86-1.46, p = 0.39) were not associated with a significant difference in the incidence of malaria compared to children who were not underweight.

**Table 3:** Variables associated with the incidence of malaria

Variable	Incidence of malaria		Unadjusted IRR* (95% CI)	p-value	Adjusted IRR** (95% CI)	p-value
					
	Exposed^†^	Unexposed				
Measures of stunting						
none	-	2.87	reference	-	reference	-
mild	4.02	-	1.55 (1.31-1.83)	< 0.001	1.24 (1.06-1.46)	-0.008
moderate-severe	3.89	-	1.64 (1.37-1.98)	< 0.001	1.24 (1.03-1.48)	0.02

Breastfeeding	2.93	4.02	0.56 (0.50-0.63)	< 0.001	0.85 (0.68-1.07)	0.17

HIV infected	2.06	3.91	0.56 (0.43-0.75)	< 0.001	0.93 (0.67-1.30)	0.68

TS chemoprophylaxis	2.18	5.05	0.38 (0.33-0.43)	< 0.001	0.50 (0.41-0.61)	< 0.001

Urban vs. rural residence	1.65	4.19	0.41 (0.31-0.52)	< 0.001	0.44 (0.34-0.56)	< 0.001

Age per 1 year increase	N/A		2.22 (2.01-2.44)	< 0.001	1.49 (1.20-1.84)	< 0.001

^† ^Exposed groups include stunted, breastfeeding, HIV infected participants, TS chemoprophylaxis, and urban residence

* *IRR *incidence rate ratio using univariate analysis including only the variable of interest

**IRR incidence rate ratio using multivariate analysis including all variables of interest listed in the table

## Discussion

This study was conducted in a cohort of young children living in an area of high malaria transmission intensity, where the incidence of malaria was over 3.6 episodes per person-year despite the use of long-lasting ITNs by all study participants and TS prophylaxis in majority of the study participants. Malnutrition was also very common, with 70% of study participants meeting criteria for mild stunting, and 38% of study participants being mildly underweight. HIV infection was associated with an increased risk of both stunting and being underweight, while breastfeeding and the use of TS prophylaxis were protective against stunting, but not being underweight.

The main objective of the study was to evaluate associations between paediatric malnutrition and the incidence of malaria by HIV status. Stunting was associated with a modest 24% increase in the incidence of malaria after controlling for potential confounding factors, but the degree of stunting did not modulate this association. In addition, the association between stunting and an increased incidence of malaria was consistent between HIV-infected and uninfected children. In contrast, there was no statistically significant association between being underweight and the incidence of malaria.

Interactions between malnutrition and HIV infection are complex and have been suggested to have negative feedback loops. Malnutrition is a significant risk factor for AIDS-related mortality and HIV-associated wasting often persists even with use of ART [29,30]. The direct effect of HIV on impaired metabolic function in absorption, storage and utilization of nutrients can translate into compromised immunity, nutrient deficiencies and increased vulnerability to infectious diseases [5]. In this study, HIV-infected children were more likely to be malnourished as compared to the HIV-uninfected children, which is in accord with the existing literature. The finding from this study that breastfeeding is protective against malnutrition is also consistent with the existing literature [31,32] and highlights the importance of breastfeeding promotion as one of the most cost-effective and feasible child survival interventions [33].

Another novel finding of this study was that children taking TS prophylaxis had a significantly lower risk of stunting. A similar finding was reported in a randomized control trial of TS prophylaxis among HIV-infected adults in Côte d'Ivoire, suggesting that TS prophylaxis may improve nutritional status by the prevention of morbidity-related malnutrition or by other indirect mechanisms [34].

Associations between measures of malnutrition and the risk of malaria appear to be complex as previous studies have reported a wide range of findings. One source of this complexity is due to differences in the indicators commonly used for malnutrition. Stunting is generally considered an indicator of chronic malnutrition, wasting generally reflects a recent and severe process, and underweight a reflection of a combination of factors. Earlier studies provide some evidence of a protective effect of stunting against malaria [8,35] and the presence of malaria in famine victims within few days of re-feeding suggested that feeding provided essential nutrients for sequestered parasites leading to recrudescent infection [9,36]. More recently in a cohort of preschool children from Senegal, those who were wasted had 67% lower odds of having at least one subsequent clinical malaria attack, whereas stunting and being underweight were not associated with clinical malaria [7]. In contrast, several studies have reported an increased risk of malaria among stunted children. In a large cross-sectional study from Ghana, children who were underweight had 67% increased odds of clinical malaria [14]. In a prospective cohort study of Gambian children, those with baseline stunting had a 35% higher risk of experiencing at least one episode of malaria during rainy season [13], yet neither wasting nor being underweight influenced susceptibility to malaria. The results presented in this report support the finding that stunting, a measure of chronic malnutrition is associated with an increased risk of malaria.

There are several hypotheses as to why chronic malnutrition may increase the risk of malaria. It is now widely known that nutrition plays an important role in modulation of anti-pathogen immunity and most studies have demonstrated a deleterious effect of malnutrition on the appropriate immune response to infection [37,38]. In addition, given that chronic malnutrition is frequently accompanied by nutritional deficiencies, it is conceivable that multiple specific nutrients may influence malaria infection and pathology [39,40].

Strengths of this study include the prospective, longitudinal study design and the assessment of HIV status. The mean of anthropometric measures of malnutrition taken over an extended period of follow-up were obtained and therefore better reflect the long-term nutritional status of a child compared to studies which rely on single measurements. In addition, malaria morbidity was analysed using an incidence measure of clinical disease, which best reflects the repeated nature of malaria in children residing in high transmission areas. Another strength is the inclusion of HIV-exposed and infected children, which has not been previously reported in studies evaluating associations between malnutrition and malaria. Limitations of this study include the lack of assessment of the temporal relationship between malnutrition and malaria. A number of studies have indicated a deleterious effect of malaria on nutritional status [41,42]. Therefore, in this study it is possible that malaria itself could have been in the causal pathway of the observed association between stunting and an increased incidence of malaria. The relatively low number of children with wasting in this study was also a limitation and precluded the ability to evaluate this measure of malnutrition as a risk factor for malaria. Finally, the presence of unmeasured confounders that could explain the observed association between stunting and an increased incidence of malaria cannot be ruled out.

## Conclusion

In this study of young children living in a high transmission area, stunting was associated with an increased risk of malaria regardless of HIV status. Of note, the incidence of malaria was high in this cohort despite the use of ITNs and TS prophylaxis in a substantial number of children. Intervention studies aimed at improving nutritional status as a means of reducing the incidence of malaria in similar epidemiological settings deserve exploration.

## Competing interests

The authors declare that they have no competing interests.

## Authors' contributions

EA, TGS, JH, HW, AK, JT, MRK and GD contributed to the study design and oversight. EA, AG, WV, SY and GD contributed to the methodology, data analysis, interpretation of the results and drafting of the manuscript. All authors read and reviewed the final manuscript. All authors read and approved the final manuscript.
